# Digital therapeutics and its role in cancer treatment management: current development and future scope

**DOI:** 10.1590/1806-9282.20240183

**Published:** 2024-07-19

**Authors:** Akhilesh Vikram Singh

**Affiliations:** 1 Graphic Era Deemed to be University, Department of Biotechnology – Dehradun, India.

The progressing era has stepped toward the modernized techniques needed in health development by applying information technology to deliver a better therapeutic intervention. This is similar to other digital apps, which connect patients' clinical reports to software that scrutinizes the patient's required improvements to prevent diseases. Therefore, this is efficiently possible through introducing and implementing digital therapeutics (DTx)^1^. Unlike other diseases/disorders, cancer cells modulate the host zone and suddenly invade other regions, irrespective of therapy. Recently, the global statistical analysis revealed that a 50% increase in prevalence is expected in the forthcoming 20 years, and this would be seen as high in female breast cancer^2^. Therefore, it is essential to choose DTx, a steadily growing interest among patients in which the device principle actively responds to patients' queries based on evidence-based clinical reports built into algorithm/software (Software as a Medical Device – SaMD that empowers timely knowledge, monitoring the symptoms and related stress behavior, its management to improve the quality of life of cancer patient and to keep track on the adverse events of anti-cancer treatments, and reduces hospitalization budget^3^).

Cancer treatment has been the world's highest-expenditure treatment strategy, which remains a challenge due to the unavailability of specific diagnostic biomarkers and effective chemotherapy drugs, showing poor solubility, stability, and limited bio-distribution due to inter-individual ethnicity variations, leading to toxic side effects, and misleading both medical practitioners and patients; however, diverse minority nations attain the least benefit of the treatment or left untreated. In several cases, cancer cells are resistant to anti-cancer drugs, and to overcome this, a scarcity of immunomodulatory drug development is observed, which might benefit patients^4,5^. To overcome the challenges and to keep a self-check, patients must be aware of using the currently available approaches like DTx, which can drive a better outcome through acting like a rehabilitation center under the guidance of a physician, providing counselling sessions to bring changes in lifestyle activities like diet, regular exercise, awareness on the risk factors associated with cancer. The DTx sets some activities and some tasks to attempt and reach the goal of achieving control of mood swings, recovering from depression, and the capability to self-reduce the side effects through yoga and meditation. The DTx gives daily feedback to patients attending the automated medication management combined with a multidisciplinary remote clinical-care team^6^.

With a rapid application of cloud computing technologies and artificial intelligence (AI) embedded as sensors in mobiles and wearable gadgets like smartwatches with inbuilt measurable digital biomarkers that touch skin through which they detect and track the changes occurring in patients and transmit the vital signs as biofeedback to the patient, they have been a very useful tool in oncology^7^. A similar trend has been observed in the case of patients with breast cancer and lung cancer. The Sidekick smartphone app was designed with inbuilt information from oncologists and consisted of daily tasks regarding patient's sleep patterns, nutrition intake, psychological mood swings, depression, stress, and meditation. The outcome was recorded for 1 month as increased knowledge, self-awareness, and post-study QoL questionnaires. Finally, high acceptability, retention, and engagement were found in cancer patients who were comfortable using DTx. A global pharmaceutical organization has partnered with the Sidekick Health app to benefit breast cancer patients to confer potential therapeutic plans^8^. Similarly, Moovcare is another DTx that focuses on a questionnaire set every week to detect relapse or complications in lung cancer patients^9^.

Although the DTx apps have significantly occupied a place in routine checkups, they will be an added advantage in the future. Still, ethically, DTx is experiencing complex issues like security, regulation, and adoption of this app in all medical sectors. It is a known fact that the FDA, HIPAA, and HITECH are regulatory departments that assure the safety of patients while applying any novel app application as digital medicine to provide reliability and effectiveness, as the DTx is still evolving in medical treatment; there could be skepticism in adopting DTx and probability of lacking clarity on its consistent requirement. Crucially, with DTx, cybersecurity issues are raised as hackers obtain patients' personal medical information that is secured; therefore, DTx organizations must secure patients' data with appropriate algorithms^1^.

With the emerging DTx, in the future, there is scope for collaboration of pharma and DTx organizations to benefit the patient community. The future pipeline involves nine leading DTx companies to bring therapeutics in other therapeutic areas with major applications in neurosciences, cardiovascular, and oncology.
